# Mucosal stromal fibroblasts markedly enhance HIV infection of CD4+ T cells

**DOI:** 10.1371/journal.ppat.1006163

**Published:** 2017-02-16

**Authors:** Jason A. Neidleman, Joseph C. Chen, Nargis Kohgadai, Janis A. Müller, Anders Laustsen, Karthiga Thavachelvam, Karen S. Jang, Christina M. Stürzel, Jennifer J. Jones, Christina Ochsenbauer, Avantika Chitre, Ma Somsouk, Maurice M. Garcia, James F. Smith, Ruth M. Greenblatt, Jan Münch, Martin R. Jakobsen, Linda C. Giudice, Warner C. Greene, Nadia R. Roan

**Affiliations:** 1 Gladstone Institute of Virology and Immunology, University of California, San Francisco, San Francisco, CA, United States of America; 2 Department of Urology, University of California, San Francisco, San Francisco, CA, United States of America; 3 Center for Reproductive Sciences, Department of Obstetrics, Gynecology and Reproductive Sciences, University of California, San Francisco, San Francisco, CA, United States of America; 4 Institute of Molecular Virology, Ulm University Medical Center, Ulm, Germany; 5 Department of Biomedicine, Aarhus University, Aarhus, Denmark; 6 Department of Medicine, University of Alabama at Birmingham, Birmingham, Alabama, United States of America; 7 Center for AIDS Research, University of Alabama at Birmingham, Birmingham, Alabama, United States of America; 8 Department of Medicine, Division of Experimental Medicine, University of California, San Francisco, San Francisco, CA, United States of America; 9 Department of Medicine, Division of Gastroenterology, San Francisco General Hospital and University of California, San Francisco, San Francisco, CA, United States of America; 10 Departments of Clinical Pharmacy, Medicine, Epidemiology and Biostatistics, University of California, San Francisco, San Francisco, CA United States of America; 11 Aarhus Research Centre for Innate Immunology, Aarhus University, Aarhus, Denmark; 12 Departments of Medicine, Microbiology, and Immunology, University of California, San Francisco, San Francisco, CA, United States of America; University of North Carolina at Chapel Hill, UNITED STATES

## Abstract

Understanding early events of HIV transmission within mucosal tissues is vital for developing effective prevention strategies. Here, we report that primary stromal fibroblasts isolated from endometrium, cervix, foreskin, male urethra, and intestines significantly increase HIV infection of CD4+ T cells–by up to 37-fold for R5-tropic HIV and 100-fold for X4-tropic HIV–without themselves becoming infected. Fibroblasts were more efficient than dendritic cells at *trans*-infection and mediate this response in the absence of the DC-SIGN and Siglec-1 receptors. In comparison, mucosal epithelial cells secrete antivirals and inhibit HIV infection. These data suggest that breaches in the epithelium allow external or luminal HIV to escape an antiviral environment to access the infection-favorable environment of the stromal fibroblasts, and suggest that resident fibroblasts have a central, but previously unrecognized, role in HIV acquisition at mucosal sites. Inhibiting fibroblast-mediated enhancement of HIV infection should be considered as a novel prevention strategy.

## Author summary

The molecular basis by which small amounts of HIV can initiate infection in the mucosa is not well understood. Here, we report that genital and rectal fibroblasts, amongst the most abundant cells of the mucosa, potently increase HIV infection of T cells. Fibroblasts are more abundant and can be more effective as infection enhancers than dendritic cells, which are thought to play a role in early HIV transmission by facilitating infection of T cells. In contrast to fibroblasts, mucosal epithelial cells, which line the mucosa, inhibit HIV infection. Our findings suggest that abrasions in the genital and rectal epithelium may increase HIV transmission in part by allowing external HIV to escape an antiviral environment to access the infection-favorable environment of the stromal fibroblasts. These studies suggest that approaches to limit fibroblast-mediated enhancement may diminish HIV transmission rates.

## Introduction

HIV is transmitted primarily by traversing mucosa. Although the efficiency of sexual HIV transmission is poor, perhaps as infrequent as 1 per 1,000 episodes of sexual encounter, drivers of epithelial disruption, such as genital ulcers, markedly increase infection risk [1, 2]. Breaches in the epithelium may increase access of HIV to sub-epithelial components, including early cellular targets such as CD4+ T cells [3]. Access to this compartment also increases viral contact with resident dendritic cells (DCs), which although inefficiently infected, can capture virions and transfer them to CD4+ T cells by *trans*-infection. Immature DCs (iDCs) patrol mucosal sites and can capture HIV on DC-SIGN, a C-type lectin, to *trans*-infect CD4+ T cells [4]. Upon infection or inflammation of the mucosa, iDCs differentiate into mature DCs (mDCs), which have a higher *trans*-infection capacity [5] and use the lectin Siglec-1 instead of DC-SIGN to mediate viral transfer [6, 7]. *Trans*-infection of CD4+ T cells is significantly more efficient than cell-free HIV infection, prompting the notion that DCs promote transmission by increasing the efficiency of T cell infection [4]. Furthermore, mDCs that migrate to lymph nodes in response to infection may fuel viral spread by forming conjugates with lymphoid CD4+ T cells [7].

Following vaginal inoculation of rhesus monkeys with SIV, viral replication initiates early, and systemic spread has already occurred after 24 h [8]. Since differentiation of iDCs into mDCs with increased ability to transfer virions requires at least 24 h [6], in mucosa without preexisting inflammation, the initial DCs to encounter HIV are likely iDCs. However, iDCs are not abundant at all mucosal portals of HIV entry. DC-SIGN+ DCs are relatively rare in the vaginal mucosa [9], and DCs appear to be absent altogether in the penile urethra [10], suggesting that DCs are not a primary player in HIV transmission at all mucosal sites.

While DCs are not abundant at all mucosal sites, stromal fibroblasts are. Fibroblasts are ubiquitous, providing much of the structural framework of tissues, and they have diverse functions ranging from regulating immunity to control of tissue remodeling [11, 12]. One of the best characterized classes of these cells are endometrial stromal fibroblasts (eSF), which are essential for implantation of the embryo and maintenance of pregnancy in humans [12, 13]. Extensive characterization of eSF has been facilitated by their abundance and availability due to the monthly regenerative potential of the endometrium. We developed protocols to cryopreserve and culture these cells [14] and showed that they retain functional properties, including responsiveness to ovarian steroids, upon *ex vivo* culture [15]. Here, we use eSF as a model to determine if primary mucosal fibroblasts influence HIV infection and compare them to epithelial cells from the same tissue. Because of the unexpectedly high capacity of eSF to enhance HIV infection, we tested fibroblasts from other HIV mucosal entry sites, finding that they too exhibit a potent ability to enhance HIV infection.

## Results

### eSF enhance HIV infection of CD4+ T cells

**Fig 1:**
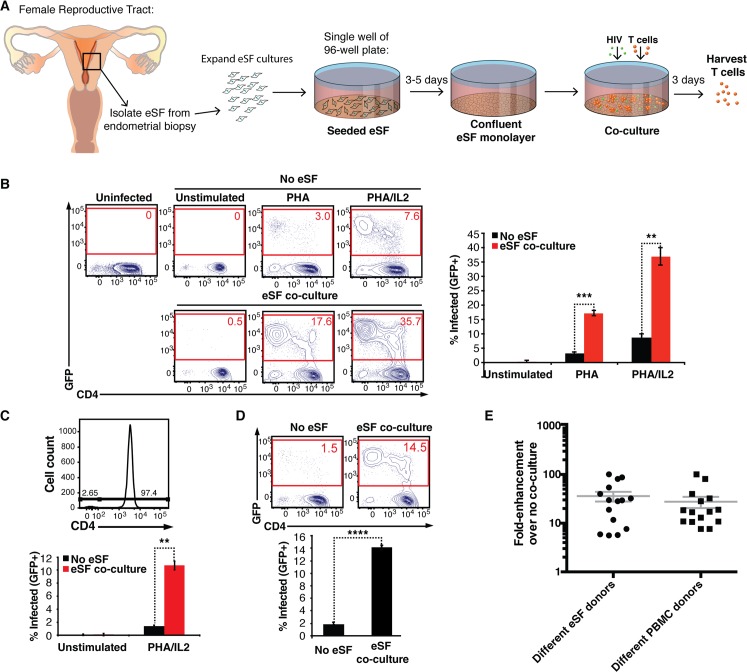
eSF enhance HIV infection of CD4+ T cells. (A) Schematic of infection of T cells in the absence or presence of eSF co-culture. eSF are grown until confluency, at which time PBMCs are added at a ratio of 10 PBMC: 1 eSF. HIV is then added and infection rates are assessed 3 d later by flow cytometry. (B) Unstimulated, PHA-activated, or PHA/IL2-activated PBMCs were infected with an HIV reporter virus encoding GFP (NLENG1I, derived from CXCR4-tropic NL4-3) in the absence or presence of eSF, and monitored 3 d later for levels of productive infection. *Left*: Representative flow cytometric plots. *Right*: Results of experimental triplicate measurements. Results are gated on live, singlet CD3+CD8- cells. **p<0.01, ***p<0.001 (by 2-tailed *t* test). Representative of data from >10 experiments each with different eSF donors. (C) Purified CD4+ T cells were infected with NLENG1I in the absence or presence of eSF. *Top*: PBMC-derived CD4+ T cells were isolated to >97% purity. *Bottom*: These cells were left unstimulated or activated with PHA/IL2, infected with NLENG1I in the absence or presence of eSF, and monitored for infection levels 3 d later by flow cytometry. Results are gated on live, singlet CD3+CD8- cells. **p<0.01 (by 2-tailed *t* test). Representative of data from 3 experiments each with different eSF donors. (D) CD3/CD28-activated PBMCs were infected with NLENG1I in the absence or presence of eSF and monitored 3 d later for infection. *Top*: Representative flow cytometric plots. *Bottom*: Results of experimental triplicate measurements. Results are gated on live, singlet CD3+CD8- cells. ****p<0.0001 (by 2-tailed *t* test). Representative of data from 3 experiments each with different eSF donors. (E) Fold-enhancement of NLENG1I infection of PBMCs when comparing infection rates in the presence versus absence of eSF. Shown are the results summarizing 15 experiments each using a different eSF donor (*left*, mean: 36-fold), and 15 experiments each using a different PBMC donor (*right*, mean: 28-fold).

To determine whether eSF, the most abundant cells in the endometrial stroma, affect the efficiency of HIV infection of permissive cells, we infected PHA-activated PBMCs with NLENG1I (a GFP reporter virus derived from NL4-3 [16]) in the absence or presence of a pure population of eSF (S1 Fig). This was accomplished by adding PBMCs to a confluent monolayer of eSF at a 10:1 ratio, immediately followed by addition of HIV. Three days later, PBMCs were harvested from the culture and assessed for levels of HIV infection by flow cytometry (Fig 1A). CD4+ T cells were infected at significantly higher rates when eSF were present (Fig 1B). This phenotype was recapitulated with purified CD4+ T cells (Fig 1C), demonstrating that the effect was mediated directly on CD4+ T cells and not indirectly by other cells in PBMCs. Infection of cells activated through the T cell receptor, using anti-CD3/CD28 conjugated beads, was similarly enhanced by eSF co-culture (Fig 1D). Tissue-derived CD4+ T cells, naturally permissive to infection without exogenous activation, were also susceptible to eSF-mediated enhancement of HIV infection (S2 Fig). The enhancement of infection rates is a general property of eSF and not donor-specific, as fold-enhancement rates from 6-100-fold were observed in 15 independent experiments each using eSF from a different donor (Fig 1E). A similar range of infection enhancement was observed when PBMCs from 15 different donors were used (Fig 1E). Therefore, although all eSF donors tested enhanced HIV infection of T cells, the extent of enhancement differed between donors.

We noticed that the highest fold-enhancement rates were observed when the baseline levels of HIV infection (in the absence of eSF) were the lowest. To determine whether lowering the viral inoculum can increase the extent of infection enhancement, we measured T cell infection rates in the presence of decreasing doses of HIV, in the absence and presence of eSF. As the concentration of input virus decreased, the fold-enhancement of infection mediated by eSF increased (S3A Fig). When the viral inoculum was diminished to the extent that infection without eSF was close to zero, fold-enhancement rates from 71-101-fold were observed (S3B Fig). Because during natural transmission the amounts of HIV present are markedly lower than those typically used to monitor infection *in vitro* [17], eSF-mediated enhancement *in vivo* may be even more potent than what is observed in these *in vitro* assays.

**Fig 2:**
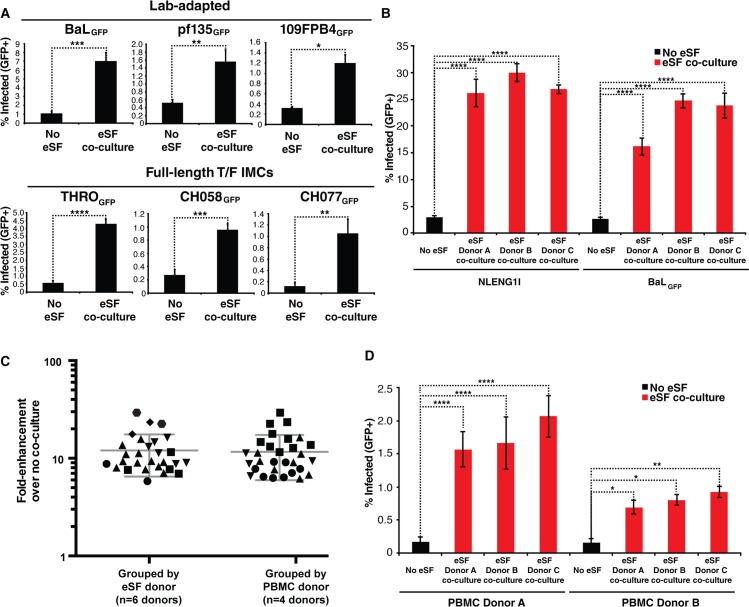
eSF-mediated enhancement of HIV infection of CD4+ T cells occurs with R5-tropic HIV, including T/F strains. (A) PHA/IL2-activated PBMCs cultured with or without eSF were infected with the indicated R5-tropic HIV and monitored by flow cytometry for infection levels 3 d later. Results are gated on live, singlet CD3+CD8- cells. *p<0.05, **p<0.01, ***p<0.001, ****p<0.0001 (by 2-tailed *t* test). (B) PHA/IL2-activated PBMCs were infected with X4-tropic NLENG1I or R5-tropic BaL_GFP_ in the absence or presence of eSF from 3 different donors, and monitored 3 d later for levels of productive infection. Results are gated on live, singlet CD3+CD8- cells. ****p<0.0001 relative to no coculture in a group-wise comparison (one-way analysis of variance with a Bonferroni posttest). (C) Fold-enhancement of BaL_GFP_ infection of PBMCs when comparing infection rates in the presence versus absence of eSF. Shown on the left are fold-enhancements mediated by six different donors of eSF, where each shape corresponds to one eSF donor (mean: 12-fold). Shown on the right are fold-enhancements mediated by four different donors of PBMCs, where each shape corresponds to one PBMC donor (mean: 12-fold). (D) PHA/IL2-activated PBMCs were infected with a T/F full-length GFP-IMC THRO_GFP_ in the absence or presence of eSF from 3 different donors, and monitored 3 d later for levels of productive infection. Results are gated on live, singlet CD3+CD8- cells. **p<0.01, **p<0.01, ****p<0.0001 relative to no coculture in a group-wise comparison (one-way analysis of variance with a Bonferroni posttest).

For eSF-mediated enhancement of HIV infection to be relevant during transmission, it would have to also occur with CCR5(R5)-tropic HIV-1, in particular transmitted/founder (T/F) virus. We therefore next tested the ability of eSF to enhance infection rates of 6 different R5-tropic reporter viruses, all engineered to express GFP upon productive infection. These 6 viruses were 2 lab-adapted strains expressing lab-adapted envelopes (BaL and pf135 env), a lab-adapted strain expressing the T/F envelope 109FPB4 [18], and 3 full-length T/F infectious molecular clone (IMC) viruses (THRO.c, CH058.c, and CH077.t) [19]. Although infection rates of the full-length IMC T/F viruses were generally low, eSF enhanced infection by all 6 R5-tropic strains (Fig 2A). We further characterized this enhancement for two of the six R5-tropic viruses, BaL_GFP_ and the T/F virus THRO_GFP_. eSF enhanced HIV infection of X4-tropic NLENG1I to a similar extent as that of BaL_GFP_ (Fig 2B). When low amounts of BaL_GFP_ were used as input, fold-enhancement rates up to 37-fold were observed (S4A Fig). Examination of eSF-mediated enhancement of BaL_GFP_ infection in 29 independent experiments with 6 different eSF donors revealed fold-enhancement rates ranging from ~6-30-fold (Fig 2C). Comparison of infection levels in experiments using different T cell donors confirmed that enhancement was not specific to particular PBMC donors (Fig 2C). Potent eSF-mediated enhancement of infection by THRO_GFP_ was also observed with multiple eSF and PBMC donors (Fig 2D). Finally, similar to eSF-mediated enhancement of NLENG1I, eSF enhanced BaL_GFP_ and THRO_GFP_ infection of purified CD4+ T cells, and efficient enhancement was observed with T cells activated through either PHA/IL2- or anti-CD3/CD28 (S4B–S4D Fig).

To ensure the phenotype was due to enhanced infection of CD4+ T cells, and not loss of bystander cells thus increasing the percentage of HIV-infected T cells, we conducted two experiments. First, we quantitated the absolute numbers of HIV-infected T cells, and found that they significantly increased in the presence of eSF (S5A Fig). Second, we infected activated PBMCs with a Firefly luciferase reporter HIV-1 and found that the luminescence signal (proportional to infection levels) was significantly increased with eSF (S5B Fig). This increase can only be caused by an increase in infection rates and not by a decreased proportion of bystander cells (since luminescence reflects the number of infected cells irrespective of the number of bystander cells), confirming that eSF increased HIV infection.

One possible explanation for how eSF enhance HIV infection is that they allogeneically activate the CD4+ T cells. To investigate this, we assessed whether CD4+ T cells cultured with eSF were more activated than those cultured without. Expression of CD25 and HLA-DR on CD4+ T cells was not altered by eSF (S6A Fig). These results are consistent with the lack of MHC class II expression on fibroblasts and their inability to stimulate allogeneic proliferation of T cells [20]. To confirm that allogeneic activation was not involved, we demonstrated that infection of activated PBMCs was increased to similar extents by autologous and heterologous eSF (S6B Fig). Interestingly, eSF also enhanced infection of unstimulated CD4+ T cells, although never in the same range as for activated cells (S6B Fig). However, when we assessed the fold-enhancement of HIV infection mediated by eSF, similar results were obtained between activated (5.3–45.0 fold) and unstimulated (5.4–45.6 fold) cells.

To assess the specificity of eSF-mediated enhancement, we tested whether HeLa cells, derived from cervical epithelium, enhance HIV infection rates. Whereas eSF enhanced infection, HeLa cells did not (S7A Fig). In contrast, T ESCs, an immortalized eSF cell line, enhanced infection to an extent similar to that mediated by primary eSF (S7B Fig). Because HIV-1 attaches to HeLa cells through cell-surface heparans [21], these results imply that the ability of adherent cells to bind HIV is not sufficient for infection enhancement.

### Mechanism of eSF-mediated enhancement of HIV infection

Because previous studies have suggested that eSF support low levels of productive infection by X4- but not R5-tropic HIV-1 [22], it was possible that productive infection of eSF increases the amount of NLENG1I available to infect co-cultured T cells. To investigate this possibility, we quantitated GFP+ eSF in eSF/T cell co-cultures exposed to HIV-1, and detected no HIV-infected eSF for both CXCR4- and CCR5-tropic HIV strains (S8A Fig). To provide further support for the notion that infection enhancement does not result from productive infection of eSF, we assessed whether eSF enhance infection of T cells by single-round virus. As with replication-competent virus, infection rates of single-round X4- and R5-tropic HIV-1 were both enhanced by eSF (S8B and S8C Fig), confirming that eSF do not enhance infection rates by producing more virions.

We next wanted to rule out the possibility that eSF were increasing the kinetics of HIV infection instead of actual infection levels. To accomplish this, we assessed the percentage and absolute numbers of HIV-infected T cells on days 1, 2, 3, 5, and 7 post-infection (S9 Fig). For both X4-tropic NLENG1I and R5-tropic BaL_GFP_, the proportion and absolute numbers of HIV-infected cells in the presence of eSF exceeded that in the absence of eSF at all timepoints examined. While the proportion of HIV-infected cells progressively increased from days 1–7, the absolute number of infected cells peaked at days 3–5 and then decreased by day 7, due to increased rates of cell death at this late timepoint. These results demonstrate that eSF-mediated enhancement of HIV infection is not the result of increased infection kinetics.

**Fig 3:**
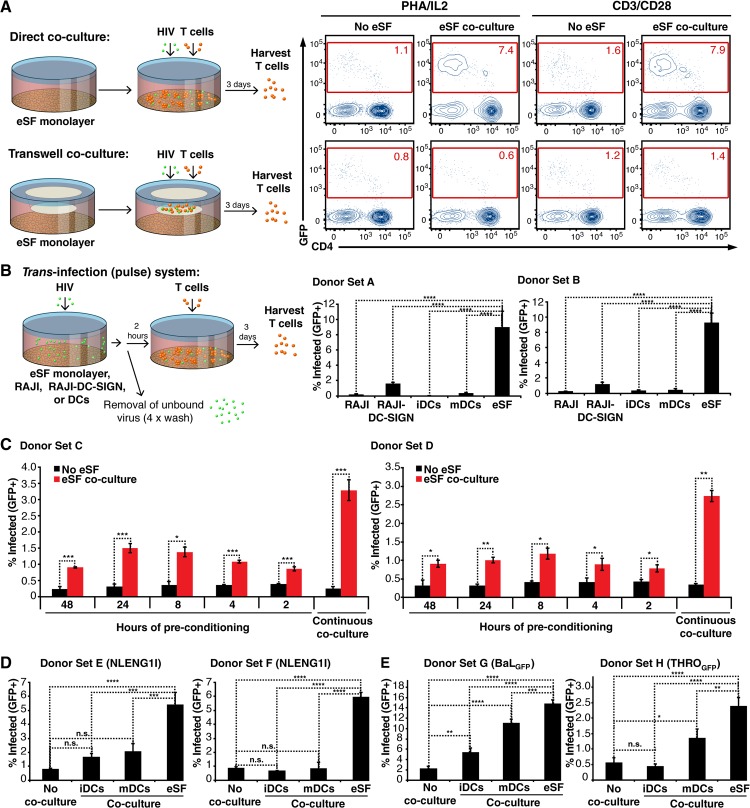
eSF enhance HIV infection more potently than DCs in a manner involving both *trans*-infection and increased susceptibility of T cells to HIV infection. (A) eSF-mediated enhancement of HIV infection requires direct contact between eSF and CD4+ T cells. PHA/IL2-activated PBMCs and eSF cultured together or separated by a transwell were infected with NLENG1I and monitored 3 d later for infection levels. The schematic on the left illustrates the regular co-culture system where cells are in direct contact, compared to the transwell system where PBMCs are physically separated from eSF. Plots in the top row correspond to direct co-culture conditions, plots in the bottom row correspond to transwell co-culture conditions. Results are gated on live, singlet CD3+CD8- cells. (B) *Left*: Schematic of *trans*-infection system where donor cells are pulsed with HIV for 2h, washed, and then added to activated T cells. *Right*: eSF pulsed with HIV transfer infectious virions to CD4+ T cells more efficiently than do RAJI, RAJI-DC-SIGN, iDCs, and mDCs. The indicated cell type was pre-treated for 2 h with NLENG1I, washed, and incubated with PHA/IL2-activated PBMCs. Infection levels were monitored 3 d later by flow cytometry. Results are gated on live, singlet CD3+CD8- cells, and are representative of three independent experiments with different sets of donors in each experiment. ****p<0.0001 relative to eSF in a group-wise comparison (one-way analysis of variance with a Bonferroni posttest). When donor cells were omitted during the HIV pulse, no detectable infectious virus was transferred to the T cells. (C) eSF enhance HIV infection of CD4+ T cells independently of *trans*-infection. PHA/IL2-activated T cells were incubated with or without eSF for 48, 24, 8, 4, or 2 h, and then removed and washed. The mock-treated or eSF-conditioned T cells were then infected with NLENG1I. Alternatively, infection was allowed to proceed in the continuous presence of eSF. Infection levels were monitored 3 d later by flow cytometry. Results are gated on live, singlet CD3+CD8- cells, and are representative of three independent experiments with different sets of donors in each experiment. *p<0.05, **p<0.01, ***p<0.001 (2-tailed *t* test). (D) PHA/IL2-activated PBMCs from three donors were infected with NLENG1I in isolation, or in the presence of eSF or an equal number of iDCs or mDCs. Infection levels were monitored 3 d later by flow cytometry. Results are gated on live, singlet CD3+CD8- cells. Shown are two out of three independent experiments with different sets of eSF and DC donors in each experiment. ***p<0.001 and ****p<0.0001 in a group-wise comparison (one-way analysis of variance with a Bonferroni posttest). (E) PHA/IL2-activated PBMCs were infected with BaL_GFP_ (*left*) or THRO_GFP_ (*right*) in isolation, or in the presence of eSF or an equal number of DCs. Infection levels were monitored 3 d later by flow cytometry. Results are gated on live, singlet CD3+CD8- cells. *p<0.05, **p<0.01, ***p<0.001 and ****p<0.0001 in a group-wise comparison (one-way analysis of variance with a Bonferroni posttest).

To define the conditions necessary for infection enhancement, we next separated eSF and lymphocytes with a transwell during co-culture. Use of a transwell abrogated enhancement (Fig 3A), demonstrating that cell contact between eSF and CD4+ T cells is necessary. We further observed that cell contact during infection increased viral fusion to CD4+ T cells (S10 Fig). As *trans*-enhancement of HIV infection requires cell contact between the donor and recipient cell [4] and increases viral fusion to cellular targets [23], these data support the possibility that eSF are capable of *trans*-infection.

To directly test whether eSF pulsed with HIV-1 could transfer HIV to CD4+ T cells, eSF were compared to DCs and RAJI-DC-SIGN, cells extensively studied for their ability to *trans*-infect T cells [4, 24]. Because iDCs and mDCs are both capable of *trans*-infection, we generated both types of DCs (S11A Fig). Equal numbers of RAJI-DC-SIGN, iDCs, mDCs, and eSF were compared for ability to transfer virus to permissive CD4+ T cells. The system used here differs from the co-culture system described thus far in that the donor cells are exposed to virus for only 2 h, washed to remove unbound virus, and then co-cultured with T cells (Fig 3B). Surprisingly, eSF could be up to >10-fold more potent than the other cell types in transferring infectious virus to T cells (Fig 3B). The different efficiencies of eSF and DCs in transferring virus to T cells could not be accounted for by virus binding, since eSF bound similar amounts of the virus as DCs (S11B Fig). Interestingly, we found that the receptors mediating *trans*-infection by iDCs (DC-SIGN [4]) and mDCs (Siglec-1 [6, 7]) are not expressed on eSF (S11C Fig), indicating that *trans*-infection in eSF differs from that of DCs and RAJI-DC-SIGN. Mannan, which binds DC-SIGN and other lectins, inhibited *trans*-infection by RAJI-DC-SIGN and iDCs but not eSF (S11D Fig), suggesting that the eSF *trans*-receptor is not a member of the mannan-binding lectin receptor family. These data suggest that eSF bind HIV in a manner not involving the main HIV-binding receptors used by DCs.

To assess whether heparin sulfate proteoglycans (HSPG), cell-surface receptors known to bind HIV-1 [25], may be responsible for eSF-mediated enhancement of HIV infection, we determined whether heparinase-treated eSF could enhance HIV infection. Pre-treatment of eSF with heparinase diminished the levels of cell-surface HSPG, but did not abrogate the ability of eSF to enhance HIV infection (S11E Fig). These results suggest that HSPG are unlikely to play a major role in eSF-mediated enhancement of HIV infection.

Having demonstrated that eSF can efficiently transfer virus to T cells, we then questioned whether eSF could also enhance HIV infection independent of *trans*-infection. Activated T cells were cultured with or without eSF for 48, 24, 8, 4, or 2 h. Lymphocytes were then separated from the eSF, washed extensively, and infected with HIV-1. Strikingly, co-culture for only 2 h rendered the T cells more permissive to infection (Fig 3C). This suggested that eSF might induce phenotypic changes in CD4+ T cells to render them more permissive to infection. However, co-culture of T cells with eSF for 3 d did not increase cell-surface levels of CD4 or the co-receptors, or the adhesion molecules LFA-1 and ICAM-1 (S12 Fig), suggesting that the increased permissivity was not due to upregulation of receptor, co-receptor, or adhesion molecules that facilitate cell-to-cell transmission.

We postulated that infection levels in eSF-conditioned T cells were lower than those of cells in continuous co-culture (Fig 3C) because in the latter condition, *trans*-infection was occurring at the same time. We reasoned that since eSF could both *trans*-infect and induce an increased state of permissivity in CD4+ T cells, they should be more effective than DCs at enhancing infection. Indeed, eSF were more effective than both iDCs and mDCs (Fig 3D). However, a 10-fold excess of DCs was sufficient to rescue enhancement to levels comparable to that mediated by eSF, and this effect was observed for both replication-competent and single-round HIV-1 (S13 Fig). Interestingly, the increased infection rates in the presence of eSF relative to iDCs and mDCs, and of mDCs relative to iDCs, could not be accounted for at the level of viral binding (S11B Fig), suggesting that viral capture by donor cells does not fully determine the efficiency of infection enhancement. When R5-tropic instead of X4-tropic HIV was used, eSF also potently enhanced infection; interestingly, however, infection enhancement by DCs was more comparable to that of eSF (Fig 3E). This was observed for both lab-adapted BaL_GFP_ and T/F THRO_GFP_. These results suggest that eSF are at least as efficient as DCs, and can be significantly more so, in promoting HIV infection of T cells.

### eSF enhance HIV infection of T cells in the presence of other mucosal components

**Fig 4:**
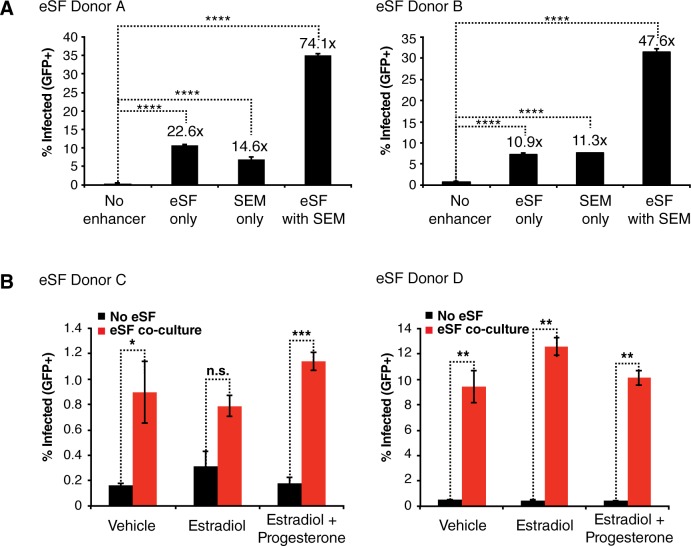
eSF enhance HIV infection of CD4+ T cells in the presence of semen fibrils and ovarian steroids. (A) Activated PBMCs were infected with BaL_GFP_ in the presence of eSF from two donors, in the presence of SEM fibrils, or in the presence of the two in combination. Infection levels were monitored 3 d later by flow cytometry. Numbers above bars correspond to fold-enhancement relative to infection levels in the absence of both eSF and SEM fibrils. ****p<0.0001 relative to No enhancer control in a group-wise comparison (one-way analysis of variance with a Bonferroni posttest). Similar results were observed with X4-tropic HIV-1. (B) eSF from two donors were treated for 10–14 d with vehicle, estradiol alone, or estradiol and progesterone in order to induce eSF decidualization. Proper decidualization of cells treated with estradiol and progesterone was confirmed by IGFBP-1 ELISA (IGFBP1 levels > 500 ng/100,000 cells in 0.5 ml culture). PHA/IL2-activated PBMCs incubated with or without vehicle- or hormone-treated eSF were infected with BaL_GFP_ and monitored 3 d later for infection levels by flow cytometry. Results are gated on live, singlet CD3+CD8- cells. Results are representative of data from four eSF donors total. *p<0.05, **p<0.01, ***p<0.001, n.s. = non-significant (2-tailed *t* test). Similar results were observed with X4-tropic HIV-1.

We next investigated whether eSF-mediated enhancement of HIV infection was relevant in situations more reminiscent of the genital mucosa. Since sexual transmission of HIV to women almost always occurs in the presence of semen, infection in the FRT should account for the effect of semen factors. We previously published that semen markedly enhances HIV infection *in vitro* [26, 27]. This property was attributed to infection-enhancing amyloid fibrils which are detected in semen from both uninfected and HIV-infected men [26, 28–30]. To determine whether eSF-mediated enhancement occurs in the presence of semen fibrils, we combined the two components, and found that eSF and semen fibrils synergistically enhanced BaL_GFP_ infection of CD4+ T cells (Fig 4A).

**Fig 5:**
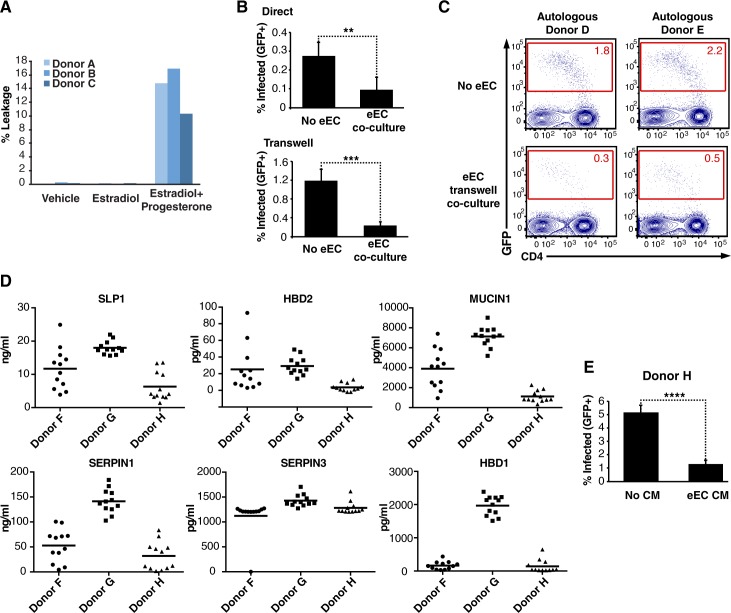
eEC inhibit HIV infection of CD4+ T cells and secrete antivirals. (A) eEC from 3 donors were co-cultured in a transwell with autologous eSF in the basolateral chamber for 6–7 days without hormones (vehicle), with estradiol only (mimicking the follicular phase), or with both estradiol and progesterone (mimicking the luteal phase). Epithelial permeability was monitored by quantitating leakage of phenol red from the apical to the basolateral chamber. (B) PHA/IL2-activated PBMCs were infected with NLENG1I with or without eEC co-culture, and monitored 3 d later for levels of productive infection. PBMCs were either in direct contact with eEC, or separated from them through use of a transwell. Results are gated on live, singlet CD3+CD8- cells, and are representative of data from three donors. **p<0.01, ***p<0.001 (by 2-tailed *t* test). (C) PHA/IL2-activated PBMCs cultured with or without autologous eEC were infected with NLENG1I and monitored for infection levels 3 d later. Results are gated on live, singlet CD3+ cells. (D) Secreted levels of the top six anti-HIV factors overexpressed in eEC relative to eSF. The levels of SLPI, HBD2, SERPINA3, MUC1, SERPINA1, and HBD1 were quantitated in the culture media of eEC from three donors (12 cultures per donor) after 48 h. (E) Activated PBMCs were infected with NLENG1I with or without eEC-conditioned media and harvested 3 d later to monitor levels of productive infection. Data are representative of three independent experiments with CM from three different eEC donors. Results are gated on live, singlet CD3+CD8- cells. ****p<0.0001 (by 2-tailed *t* test).

HIV transmission through the female reproductive tract (FRT) is influenced by estradiol and progesterone [31]. To determine whether eSF-mediated enhancement occurs with these steroids, we compared eSF treated with or without estradiol (to model the proliferative phase) or estradiol and progesterone (to model the secretory phase), and found that hormone-treated eSF were not diminished in their ability to enhance BaL_GFP_ infection (Fig 4B). We recently established an *ex vivo* dual-chamber model system where polarized endometrial epithelial cells (eEC) are cultured in a transwell with patient-matched eSF in the bottom chamber [14]. Interestingly, using this model, we found that estradiol and progesterone, but not estradiol alone, increased the permeability of an intact eEC monolayer (Fig 5A). These data are consistent with those previously reported [32] and indicate that ovarian steroid conditions characteristic of the endometrial secretory phase do not inhibit eSF-mediated enhancement of HIV infection, and may even increase access of HIV to the infection-enhancing effects of eSF by diminishing eEC barrier function.

### eEC do not enhance HIV infection of CD4+ T cells

If breaches in the genital epithelium facilitate HIV transmission by providing the virus access to infection-promoting fibroblasts, then under non-breached conditions HIV infection rates should not be enhanced. When the endometrial epithelium is intact, luminal HIV virions primarily encounter eEC. As such, we hypothesized that eEC, unlike eSF, should not be efficient enhancers of HIV infection. To test this, we infected CD4+ T cells with NLENG1I in absence or presence of primary eEC, under conditions matching those used for eSF co-culture. Not only did eEC not enhance infection, they potently inhibited it (Fig 5B and 5C). This effect did not require direct contact between T cells and eEC (Fig 5B) and occurred with autologous T cells (Fig 5C).

To better understand how eSF and eEC exert opposing effects on T cell susceptibility to HIV infection, we compared global gene expression patterns in the two cell types by microarray analysis (n = 4 donors) (S1 Table). The most differentially expressed gene in eEC vs. eSF (211-fold difference) was secretory leukocyte peptidase inhibitor (SLPI), a secreted inhibitor of serine proteases with anti-HIV activity [33]. SLPI may disrupt early viral entry by impairing annexin II-mediated stabilization of viral fusion [34]. The second most differentially expressed gene was β-defensin 4 (DEFB4A, or HBD2), which was expressed 171-fold higher in eEC. HBD2 also has anti-HIV activity and limits infection through multiple mechanisms including direct viral inactivation and restriction of reverse transcription [35]. Other antiviral genes that were highly overexpressed in eEC versus eSF include β-defensin 1 (DEFB1, or HBD1), SERPINA1, SERPINA3, and MUC1 [35–37]. All six factors were secreted into eEC culture supernatants, although levels varied between donors and different cultures from the same donor (Fig 5D). Conditioned media from eEC cultures inhibited HIV infection of CD4+ T cells (Fig 5E), suggesting that the inhibitory effects of eEC are indeed mediated by soluble factors. This finding is consistent with a prior study reporting the ability of eEC conditioned media to inhibit HIV infection of the TZM-bl reporter cell line [38]. These data provide a molecular explanation for why eEC, in contrast to eSF, inhibit HIV infection of CD4+ T cells under similar co-culture conditions. Furthermore, the data support a model whereby the luminal eEC environment is hostile for HIV, whereas the eSF-rich stromal compartment may constitute a more favorable environment for HIV replication.

### Ability to enhance HIV infection of CD4+ T cells is a general property of mucosa-associated fibroblasts

**Fig 6:**
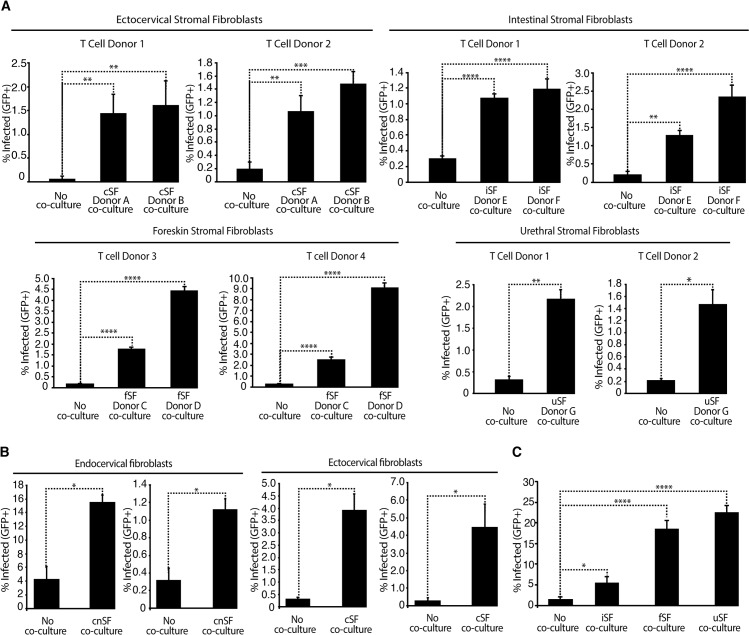
Fibroblasts from the ectocervix, intestine, foreskin, and male urethra enhance HIV infection of CD4+ T cells. (A) PHA/IL2-activated CD4+ T cells from four donors (labeled 1–4) cultured in the absence or presence of the indicated fibroblasts were infected with NLENG1I and monitored for infection levels 3 d later by flow cytometry. Results are gated on live, singlet CD3+CD8- cells. *p<0.05, **p<0.01 relative to no co-culture in a group-wise comparison (one-way analysis of variance with a Bonferroni posttest). (B) PHA/IL2-activated CD4+ T cells were infected with BaL_GFP_ in the absence or presence of cnSF from two different donors (*left*) or cSF from two different donors (*right*) and monitored for infection levels 3 d later by flow cytometry. Results are gated on live, singlet CD3+CD8- cells. *p<0.05 by 2-tailed *t* test. (C) PHA/IL2-activated CD4+ T cells were infected with BaL_GFP_ in the absence or presence of iSF, fSF, or uSF and monitored for infection levels 3 d later by flow cytometry. *p<0.05, ****p<0.0001 relative to no co-culture in a group-wise comparison (one-way analysis of variance with a Bonferroni posttest).

Finally, we wanted to broaden our investigations to determine whether stromal fibroblasts from other mucosal portals of HIV entry could enhance infection. Ectocervical stromal fibroblasts (cSF) significantly enhanced HIV infection (Fig 6A), and a comparison of eSF, cSF, and endocervical stromal fibroblasts (cnSF) from the same subject demonstrated that these cell types enhanced HIV infection similarly (S14 Fig). During transmission to men, the foreskin, male urethra, and gastrointestinal tract are potential initial sites of HIV entry. Like fibroblasts from the FRT, those from intestines (iSF), adult foreskin (fSF), and urethra (uSF) all significantly enhanced HIV infection (Fig 6A). Finally, we confirmed that the ability of these other mucosal fibroblasts to enhance HIV infection of T cells is also observed with R5-tropic HIV (Fig 6B and 6C). These observations imply that breaches in genital and gastrointestinal epithelia can facilitate transmission by exposing HIV to a fibroblast-rich environment that promotes infection.

## Discussion

**Fig 7:**
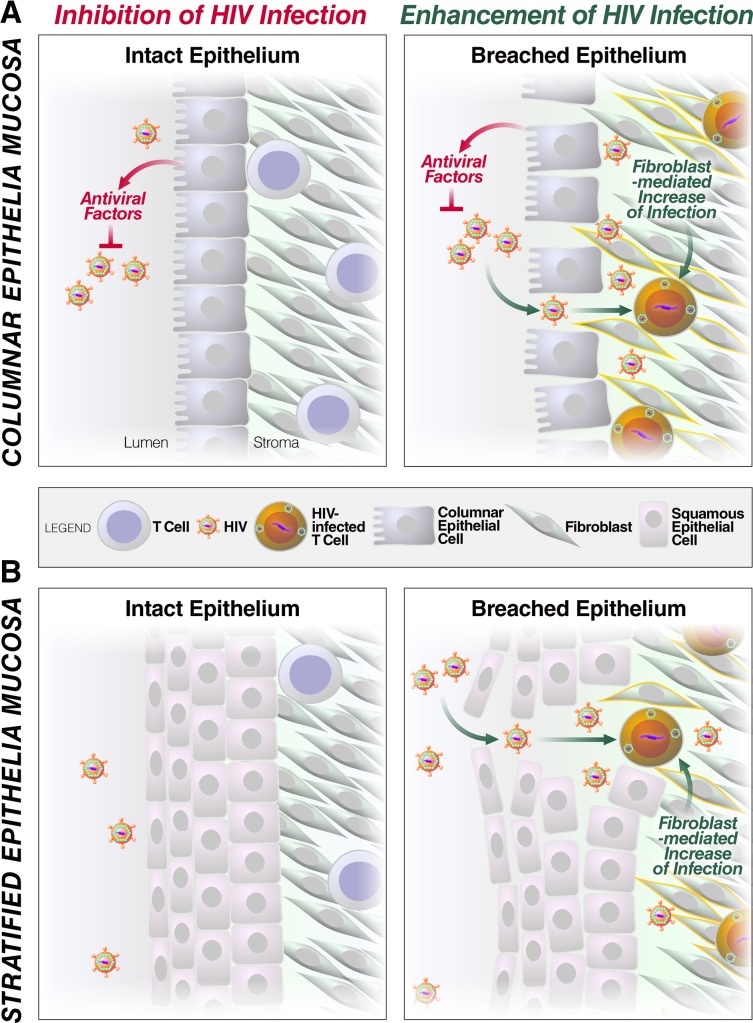
Model of fibroblast-mediated enhancement of HIV infection at mucosal portals of entry. (A) In mucosal tissues lined by single-layered columnar epithelia, such as the endometrium, when barrier function is intact, antiviral factors secreted by the epithelium limit HIV infection. Breaches in the epithelium caused by physical trauma, ulcerative sexually transmitted diseases, or the effects of progesterone facilitate migration of HIV into the stromal compartment. This compartment can be exposed to antiviral factors, either through entry from the lumen or through basolateral secretion of these factors by the epithelial cells, but is also replete with fibroblasts which can enhance HIV infection of resident CD4+ T cells. (B) Breaches in mucosal tissues lined by multi-layered squamous epithelia (e.g. ectocervix and foreskin) similarly lead to increased likelihood of transmission due to increased access to fibroblasts.

Here we report that stromal fibroblasts from multiple mucosal portals of HIV entry markedly enhance HIV infection of CD4+ T cells. Fibroblast-mediated enhancement is extremely potent, particularly under low viral inoculum conditions, which better reflect *in vivo* transmission. Fibroblasts enhanced infection by both X4-tropic (up to 100-fold) and R5-tropic (up to 37-fold) virus. We propose a model whereby fibroblasts have a key role in HIV transmission across a breached epithelium (Fig 7). At mucosal sites lined by an intact single layer of columnar epithelium (e.g. endometrium) (Fig 7A), antiviral factors apically secreted by luminal epithelial cells limit HIV infection of cellular targets. However, when the epithelium is breached, HIV accesses the stroma where fibroblasts enhance infection of resident CD4+ T cells. Fibroblasts isolated from the ectocervix and foreskin also enhance HIV infection, suggesting that this model is also relevant in mucosa lined by stratified squamous epithelia (Fig 7B).

DCs are present in genital tissues [39], and may play a key role in initial HIV transmission due to their ability to enhance HIV infection of T cells [40], much like the model we propose for fibroblast-mediated enhancement of infection. However, several important differences exist between DCs and fibroblasts with regard to their potential roles in transmission. Within mucosal tissues, fibroblasts are more abundant than DCs, which are sometimes infrequent or absent altogether [9, 10]. Furthermore, in the absence of pre-existing inflammation, mDCs tend to be less abundant than iDCs, which are less efficient *trans*-enhancement mediators than their mature counterparts [5] (S13 Fig). Converting iDCs to mDCs requires stimulation by pathogen-associated molecular patterns (PAMPs) and/or inflammatory cytokines, whereas fibroblasts are efficient enhancers without external environmental stimulation. As shown here, on a per-cell basis, fibroblasts are significantly more efficient than iDCs and mDCs at enhancing HIV infection. It should be noted, however, that it is possible that under other experimental conditions DCs may enhance infection as well as or even more efficiently than eSF. A second important distinction between fibroblasts and DCs is that the latter are professional antigen-presenting cells and can activate naïve HIV-specific T cells [41]. By using fibroblasts instead of DCs as a vehicle for *trans*-enhancement, HIV might better avoid detection by the adaptive immune system. On the other hand, DCs, unlike fibroblasts, are migratory and can propagate infection from the mucosa to draining lymph nodes. Thus, we propose that fibroblasts enhance localized infection at the earliest stage of transmission, while DCs are important after an inflammatory response has been initiated, both to promote viral replication in the tissue but also to facilitate systemic spread of the virus.

To our knowledge, we are the first to describe *trans*-infection by fibroblasts. However, other non-hematopoietic cells have been reported to transfer virus to target cells. Genital epithelial cells can transfer HIV to permissive cells [25, 42]. However, the studies reporting this did not measure *trans*-enhancement, but rather just the ability of epithelial cells pulsed with HIV to infect permissive cells. Our studies suggest primary eEC in fact inhibit infection of CD4+ T cells, likely due to the antiviral factors they secrete. In comparison, endothelial cells both *trans*-infect and *trans*-enhance HIV infection [43]. The underlying mechanism is different from that of fibroblasts, because it requires MHC class II-dependent allogeneic activation of resting CD4+ T cells by the endothelial cells, whereas fibroblasts enhance HIV infection of autologous CD4+ T cells. Interestingly, endothelial cells promote not only productive but also latent infection [43]. It is intriguing to speculate that fibroblasts may also promote latent infection, since the latent reservoir is thought to reside largely in resting T cells, and we found that fibroblasts enhanced infection of both activated and unstimulated T cells. Because latency is likely established in tissues within days of viral exposure [44], we speculate that early interactions among fibroblasts, HIV, and T cells may lead to the establishment of a stable latent reservoir.

The breaches that facilitate HIV entry into the fibroblast-rich stromal compartment may be caused by different means, such as intercourse itself [45], ulcerative sexually transmitted diseases [46, 47], or HIV, which through induction of TNF-α can diminish eEC barrier integrity [48]. In addition, estradiol with progesterone can diminish barrier function of epithelial cells from the upper FRT. While one study reporting this attributed the effect to the activity of estradiol, another attributed it to the activity of progesterone [32, 49]. The reason for the discrepancy is not clear, but may involve differences between cell lines and primary cells. Using a primary eEC/eSF co-culture system that supports paracrine signaling [14], which affects the ability of epithelial cells to respond appropriately to sex steroids [50], we confirm that progesterone and estradiol, but not estradiol alone, diminish eEC barrier function. Progesterone and progestins (synthetic mimics of progesterone) may also induce thinning of the epithelium in the lower FRT [51], suggesting that the entire FRT may be subject to progesterone-induced permeability, which could help explain the association of progesterone/progestin levels with HIV/SIV transmission risk [52, 53]. Interestingly, after vaginal inoculation of rhesus monkeys with SIV, infected cells can be detected in the endometrium within 48 h [8, 54]. Whether animals with evidence of early endometrial infection had increased frequency of abrasions in the eEC layer was not examined, but would be interesting to explore in light of data presented here.

The mechanism used by fibroblasts to enhance HIV infection is distinct from that of DC-mediated *trans*-enhancement, as it does not involve DC-SIGN or Siglec-1. Interestingly, *trans*-infection from iDCs to CD4+ T cells is mediated through actin-rich dendrites and requires actin stabilization and inhibition of HIV endocytosis [55]. Whether similar intracellular signaling pathways are responsible for fibroblast-mediated enhancement would be a worthwhile area of future investigation. Additional studies are also required to identify the receptor used by fibroblasts to capture HIV. Although we have not ruled out HSPG as the receptor, these cell-surface components are unlikely to be the major player necessary for infection enhancement, since their removal with heparinase did not significantly affect fibroblast-mediated enhancement of HIV infection. Furthermore, there also exists a mechanism of fibroblast-mediated enhancement of infection rates independent of *trans*-infection, since eSF-conditioned T cells have increased susceptibility to HIV infection. A better understanding of the molecular basis of fibroblast-mediated enhancement of HIV infection can lead to novel prevention approaches. For example, one can envision that a combination microbicide targeting both the virus and the ability of mucosal fibroblasts to enhance HIV infection may be more effective than a single-component microbicide targeting only the virus. On a more fundamental level, the study herein reveals how HIV can exploit the environment of mucosal tissues to establish a beachhead of infection with only limiting amounts of virus, and may help explain the remarkable success of HIV at spreading predominantly as a sexually transmitted pathogen.

## Materials and methods

### Endometrial and cervical cell culture

Endometrial and cervical tissues were obtained from UCSF and the Cooperative Human Tissue Network (CHTN) (IRB # 10–07286 and 14–15361). Subjects were women between the ages of 18–49 years undergoing benign gynecologic procedures and confirmed not to be pregnant. Exclusion criteria were subjects that were HIV-infected; subjects with blood and coagulation disorders; subjects on androgen therapy, gonadotropin-releasing hormone agonists, or any form of progestins; subjects with breast, endometrial, ovarian, or fallopian tube cancer; subjects with hyperlipidemia, hypercholesterolemia, heart disease, gall bladder disease, or liver disease. After written informed consent, endometrial biopsies were obtained using a pipelle endometrial suction curette (Cooper Surgical) or by hysterectomy. Ectocervical and endocervical tissues were isolated from hysterectomy samples by dissection.

Endometrial stromal fibroblasts (eSF) and endometrial epithelial cells (eEC) were cultured as described [14, 56]. Briefly, endometrial tissues were cut into 1 mm x 1 mm pieces and digested for 2 h at 37°C with Digestion Buffer, which consisted of 6.4 mg/ml collagenase type I (Worthington Biochemical Corporation) with 100 U/ml hyaluronidase (Sigma-Aldrich) in HBSS with Ca++ and Mg++ (Life Technologies). Digested material was then run through 40-μm cell strainers (Fisher Scientific) to separate single cells from fragments of endometrial sheets and glands. The flow-through was centrifuged, washed, resuspended in serum-containing fibroblast growth medium (SCM: 75% phenol red-free DMEM/25% MCDB-105 supplemented with 10% charcoal-stripped FBS and 5 μg/ml insulin), and cultured. Cells were replaced with fresh SCM every 2–3 d during culture, and were used between passages 2–4. In some experiments, eSF were cryopreserved prior to use. Cryopreservation does not affect the cytokine profiles or decidualization capacity of eSF [14, 15], or their ability to enhance HIV infection of T cells. Imaging of eSF was conducted by bright field microscopy using an Olympus CKX41 microscope with a QIClick CCD Camera and QCapture Suite Plus software (QImaging). eEC were obtained from the digested epithelial fragments that were retained on the 40-μm cell strainers. These fragments were collected, centrifuged, washed, and cultured in KSFM (Gibco) in 1-μm transwell hanging inserts (EMD Millipore) coated with Matrigel (BD Biosciences) and cultured with patient-matched eSF in a dual-chamber system as described [14], or cultured in 24-well Matrigel-coated plates (VWR) in isolation as described [56].

Ectocervical (cSF) and endocervical (cnSF) stromal fibroblasts were isolated from the corresponding cervical tissues by first cutting the tissue into 5 mm x 5 mm pieces and then digesting them for 6 h at 37°C with 6.4 mg/ml collagenase type I. The partially digested tissue was then centrifuged and resuspended in Digestion Buffer and incubated for an additional 12 h at 25°C. Digested material was then run through 40-μm cell strainers (Fisher Scientific), after which the strainer was flipped and the retenante washed with SCM onto a culture dish. Adherent cSF and cnSF were replenished with fresh SCM every 2–3 d during culture, and used between passages 1–3.

### Hormone treatment of eSF and confirmation of decidualization

eSF were treated with ovarian steroids as described [57]. This treatment protocol allows eSF to differentiate appropriately in response to estradiol and progesterone [15, 57], including proper induction of decidualization, the progesterone-driven differentiation of eSF during the secretory phase in preparation for implantation. Briefly, cells were treated with 0.1% ethanol as vehicle control, 10 nM estradiol (Sigma-Aldrich), or 10 nM estradiol and 1 μM progesterone (Sigma-Aldrich) in SCM made with 2% instead of 10% FBS. Cells were treated for 14 d, with feeding every other day. Decidualization of cells in the presence of estradiol and progesterone was confirmed by a human IGF-binding protein 1 (IGFBP1) ELISA kit (Alpha Diagnostics International and Abcam). Samples treated with estradiol and progesterone effectively decidualized as assessed by secretion of IGFBP-1 (level > 500 ng/100K cells in 0.5 ml of culture) [15].

### Isolation and culture of fibroblasts from foreskin, intestinal, and urethral tissues

Foreskin stromal fibroblasts (fSF) were obtained from UCSF (IRB # 10–01359) or purchased from Promocell or ATCC. UCSF foreskin tissues were from adult men confirmed to be HIV-negative. To isolate fSF, tissues were cut into 5 mm x 5 mm blocks and digested overnight at 4°C with 2.4 U/ml of Dispase II (Sigma-Aldrich) in RPMI-1640 medium, after which the epidermis was separated from the dermis by peeling. The dermal layer was then digested for 2 h at 37°C with RPMI-1640 supplemented with 2 mg/ml collagenase type IV-S (Gibco) and 200 U/ml DNase I (Promega). The tissue pieces were then dissociated by trituration and passed through 70-μm cell strainers (Fisher Scientific). Cells were then pelleted, washed, and cultured in D10 media (DMEM containing 10% fetal bovine serum (FBS), penicillin (50 U/ml), streptomycin (50 μg/ml), and L-glutamine (2 mM)), and used between passages 1–3.

Intestinal stromal fibroblasts (iSF) were obtained from UCSF (IRB # 10–01218) or purchased from Lonza. UCSF intestinal biopsy donors were low-risk HIV/HCV-negative individuals at low risk for exposure and otherwise without significant comorbidities. For each patient, colorectal biopsy specimens were obtained 10–25 cm from the anal verge using jumbo forceps. Biopsy pieces were washed twice with PBS, and then transferred to RP10 medium (RPMI-1640 medium containing 10% FBS, penicillin (50 U/ml), streptomycin (50 μg/ml), and L-glutamine (2 mM)). iSF were isolated by washing the intestinal tissue twice with PBS and digesting the pieces overnight in Dispase II as described for foreskin tissue processing, except that the cells were washed and cultured in SmBM medium with SmGM-2 supplements and growth factors (Lonza). Cells were replenished with new medium every other day and used between passages 1–3.

Urethral stromal fibroblasts (uSF) were obtained from urethral tissue from a subject diagnosed with gender dysphoria and undergoing elective gender reassignment at UCSF (IRB # 10–01359). The subject was on estrogen and spironolactone (an anti-androgen), and tissue was removed as part of Gender Affirming Surgery (vaginoplasty with penile inversion and creation of a neovaginal cavity). Tissue was processed as described for the foreskin tissues, except that the cells were cultured in D10. uSF were replenished with fresh media every other day and used at passage 1.

### T and monocytic cell isolation

Primary PBMCs (obtained from the Stanford Blood Bank) were isolated from Trima reduction chamber buffy coats by Ficoll-Hypaque density gradients and cultured in RP10. In some cases, peripheral blood was obtained from endometrial biopsy donors using 7.5% EDTA as an anticoagulant. Where indicated, CD4+ T cells were purified from PBMCs by first depleting monocytes through negative selection with CD14+ microbeads, followed by positive selection with CD4+ microbeads (Miltenyi). To activate T cells, PBMCs or purified T cells were stimulated for 2 d with 100 IU/ml IL-2 (Life Technologies) in the presence of 10 μg/ml PHA (Sigma). Where indicated, anti-CD3/CD28 Dynabeads (Life Technologies) were used instead of PHA for stimulation. Cells were then isolated, washed, and used in infection assays. All activated T cells were maintained in media containing 20 IU/ml IL-2.

Tonsillar T cells were used as a source of primary T cells naturally permissive to HIV infection. Human tonsils obtained from the CHTN were minced, passed through a 40-μm cell strainer, and lymphocytes were isolated by Ficoll-Hypaque density gradient centrifugation. To enrich for CD3+ T cells, B cells were depleted with CD19+ microbeads (Miltenyi).

To generate monocyte-derived dendritic cells, monocytes from PBMCs were positively selected with CD14+ microbeads and then cultured for 6 d in RP10 supplemented with 25 ng/ml interleukin-4 (R&D Systems) and 50 ng/ml granulocyte-macrophage colony-stimulating factor (Biosource). DCs were left in this media for an additional 2 d to generate immature dendritic cells (iDCs), while mature dendritic cells (mDCs) were generated by treating the cells for 2 d with 100 ng/ml LPS (Sigma). Maturation status was confirmed by assessing cell surface expression of CD83 (see flow cytometry section).

### Cell lines

Cell lines used in this study were 293T (ATCC), HeLa (ATCC), T ESCs (ATCC), RAJI (NIH AIDS reagent), or RAJI stably expressing DC-SIGN (RAJI-DC-SIGN, NIH AIDS reagent). Hela and 293T were cultured in D10, T ESCs were cultured in SCM, and the RAJI and RAJI-DC-SIGN were cultured in RP10.

### Virus preparation

HIV-1 viruses were produced in 293T cells by Fugene-mediated transfection (Roche) or calcium phosphate transfection [58] with proviral DNA expression plasmids. All GFP viruses used were reporter viruses where GFP is expressed only in productively infected cells. The replication-competent X4-tropic GFP reporter virus used in this study was CXCR4-tropic pNL4-3 (NLENG1-IRES or NLENG1I [16], gift of David N. Levy, NYU). The R5-tropic GFP reporter viruses used in this study were as follows: BaL_GFP_ (short for pNL-GFPm.6ATRi-BaL.ecto), 109FPB4_GFP_ (short for pNL-GFPm.6ATRi-C.ZM109FPB4.ecto), pf135_GFP_ (short for pBRNL43_005pf135(R5)nef+_IRES_eGFP), THRO_GFP_ (short for pBR_THRO.c.IRES-GFP), CH058_GFP_ (short for pBR_CH058.c.IRES-GFP), and CH077_GFP_ (pBR_CH077.c.IRES-GFP). BaL_GFP_ was generated by modifying the previously described pNL-LucR.6ATRi-BaL.ecto *Renilla* luciferase (LucR) reporter proviral construct [59], in which the ectodomain of gp160/Env is encoded by the BaL *env* sequence (GenBank entry AY426110) and Nef expression is driven by a modified IRES element (referred to as 6ATRi). The LucR gene was replaced with the 720 bp coding region for monomeric EGFP.A206K (GFPm) [60] (corresponding to, and PCR amplified and cloned, from nt 4891–5607 in Genbank accession # KP202880.1, [61]). To generate 109FPB4_GFP_, the heterologous Env ectodomain-coding region derived from the T/F HIV-1 subtype C strain ZM109F.PB4 ([18] corresponding to nt 123–2020 of GenBank accession number AY424138.2) was cloned in place of the BaL *env* sequence of the BaL_GFP_ reporter construct. pf135_GFP_ is a lab-adapted CCR5-tropic HIV-1 (pBRNL43_005pf135(R5)nef+_IRES_eGFP), which was derived from the pBRNL43_nef+IRES_eGFP clone [62] where the gp120 V3 loop sequence was replaced with that of the CCR5-tropic 005pf135 HIV primary isolate as described [63]. The full-length T/F IMC THRO GFP reporter (THRO_GFP_) was engineered by cloning the proviral DNA of THRO [19] into the truncated pBR322 vector of HIV-1 NL4-3 [64] using conserved *NotI* and *MluI* sites flanking the LTRs to generate the construct pBR_THRO.c. A nef-MCS-LTR construct was generated which incorporated a multiple cloning site (MCS) consisting of two unique restriction sites (*PmeI* and *SacII*) after the *nef* stop codon, and duplicated the U3-region that overlaps with *nef* after the two restriction sites. A PCR product from nef-MCS-LTR was cloned into pBR_THRO.c, and then an IRES-eGFP PCR product generated from pIRES2-eGFP (Clontech) was cloned into the MCS to generate the THRO reporter virus. The full-length T/F IMC CH058 and CH077 GFP reporter viruses (CHO58_GFP_ and CHO77_GFP_, respectively) were generated in an analogous fashion starting with the proviral DNA sequences previously described [19]. The replication-incompetent single-round GFP reporter virus used in the eSF co-culture assays was generated by co-transfection of 293T cells with pBR-NL43-env*nef+vpr+vpu+–IRES–eGFP [65] together with NL4-3 env (pCAGGS NL43-env C-AU1) [66], and was referred to as NL4-3.env*GFP. This env-deficient NL4-3 reporter construct contains an early stop codon in *env*, and a *nef* gene followed by IRES-eGFP [67]. For single-round infection with R5-tropic HIV with or without eSF, env-pseudotyped Nef-deficient viruses were produced by transfecting 293T cells with a packaging system consisting of three plasmids: 1) CSGW, an HIV-1 vector expressing eGFP under the control of LTR [68], 2) pCRVgag/pol (courtesy of Stuart Neill, Kings College, London), and 3) pSVIII_env expressing the CCR5-tropic env from YU2 [69] (courtesy of Joseph Sodroski, Dana-Farber Cancer Institute). For viral fusion assays, provirus was co-transfected with pCMV-BlaM-Vpr and pAdvantage (Promega) as described [70] to generate BlaM-containing virus. At 48 h post-transfection, 293T supernatants containing virus were clarified by low-speed centrifugation and concentrated by ultracentrifugation at 72,000 x G for 90 min at 4°C. The HIV-1 luciferase reporter virus (NL4-3.Luc) was generated from pBRNL4-3_F-*Luc* which encodes the NL4-3 provirus with a firefly luciferase cassette in place of *nef* [71], and was used in unconcentrated form due to high potency of these viral preparations. Viral titers were measured by the Lenti-X p24^Gag^ Rapid Titer Kit (Clontech). For co-culture experiments, viruses were used at final concentrations of 15–100 ng/ml p24^Gag^.

### Fibroblast co-culture infection assays

To test the effect of primary fibroblasts on HIV infection of CD4+ T cells, fibroblasts were grown in 96-well flat-bottom tissue culture plates (Falcon) until confluency. Because the fibroblasts are confluent at the time of co-culture, their cell numbers remain constant over the course of the 3-day infection (S15 Fig). Fibroblasts were replenished with fresh media 24 h before co-culture. The indicated HIV reporter virus (15–100 ng/ml p24^Gag^) was added to resting or activated PBMCs (10^5^/well) in the absence or presence of fibroblast (~10^4^/well when confluent) such that the ratio of PBMC:fibroblast was 10:1. Cells were infected for 3 d unless otherwise indicated. In this experimental protocol, input viral inocula were not washed away prior to harvest. Where indicated, purified activated CD4+ T cells, or autologous CD4+ T cells from fibroblast donors, were used as target cells. In some experiments, lymphocytes were separated from the fibroblasts through use of 1-μm transwells (Millipore). Where indicated, fibroblasts were pre-treated for 2 h at 37°C with 1 international unit (iU) / ml of heparinase I and heparinse III from *Flavobacterium heparinum* (Sigma-Aldrich) or media alone prior to co-culture. On the day of harvest, which was 3 d post-infection unless otherwise indicated, cells were stained and assessed for infection levels by flow cytometry. Live, CD4+ T cells were analyzed through a sequential gating strategy that defined these cells as viable (as assessed through use of the Zombie Aqua amine-reactive dye from Biolegend), singlet, CD3+ CD8- cells (S16 Fig). Productively infected CD4+ T cells can be identified as CD3+ CD8- cells that have downregulated CD4 (downregulation of CD4 is mediated by HIV-1 accessory genes). Of note, donor-dependent differences in the primary fibroblasts and T cells led to variability in the % of infected cells between different experiments.

To compare the effects of eSF and HeLa on HIV infection of CD4+ T cells, confluent cultures of eSF or HeLa were tested in parallel, with HeLa cells being cultured in D10 instead of SCM. To compare viral infection enhancement mediated by eSF to that mediated by cells of monocytic lineage, equal numbers of iDCs, mDCs, RAJI, RAJI-DC-SIGN, and eSF were compared for ability to enhance HIV infection of activated CD4+ T cells as described above, except that the monocytic lineage cells were cultured in RP10. No differences in HIV infection rates were observed in the different media used (SCM, D10, RP10). Where indicated, 10x more iDCs, mDCs, RAJI, or RAJI-DC-SIGN than eSF were tested.

To test the effect of primary fibroblasts on HIV fusion to CD4+ T cells, 50 ng/ml BlaM-containing virus was added to PBMCs in 100 μl RP10, in the absence or presence of eSF. Viral fusion was allowed to proceed for 4 h, after which cells were loaded overnight with CCF2/AM dye (Invitrogen). Viral fusion was then assessed as described [70].

To test the combined effects of primary fibroblasts and semen fibrils on HIV infection of CD4+ T cells, PBMCs were stimulated with PHA/IL2. Virions were incubated in the absence or presence of 100 μg/ml SEM1(86–107) fibrils [29] for 10 min, and then added to the activated PBMCs in the absence or presence of eSF. After 3 d, the levels of infection were assessed by flow cytometry as described above.

### *Trans*-infection and HIV binding assays

To test the ability of fibroblasts to *trans*-infect CD4+ T cells, eSF were pulsed with 1 μg/ml p24^Gag^ NLENG1I for 2 h at 37°C and then washed 4x with medium. Equivalent numbers of iDCs, mDCs, RAJI, and RAJI-DC-SIGN were treated in parallel in a similar fashion by centrifuging the cells in V-bottom plates in between washes. Where indicated, mannan (50 μg/ml) was incubated with the cells for 30 min prior to viral pulse. Pulsed cells were incubated with activated PBMCs at a ratio of 1 pulsed cell:10 PBMCs. Where indicated, 10x as many pulsed iDCs and mDCs were added. Infection levels were monitored 3 d later by flow cytometry. To measure viral binding to cells, triplicate wells, each containing 10^4^ eSF, mDC, or iDC, were each pulsed with 1.5 ng p24^Gag^ of NLENG1I in a volume of 0.2 ml for 2 h at 37°C, washed 4x with medium, and then lysed for analysis by the Lenti-X p24^Gag^ Rapid Titer Kit (Clontech) to quantitate levels of cell-associated p24^Gag^.

### Pre-conditioning of T cells by fibroblasts

To test whether fibroblasts enhance the susceptibility of CD4+ T cells to HIV infection, PBMCs were activated for 2 d with 10 μg/ml PHA and 100 IU/ml IL-2 in RP10. The cells were then washed, resuspended in SCM containing 20 IU/ml IL-2, and at each time point (48, 24, 8, 4, or 2 h) cells were taken from the activated pool and in triplicates diluted 1:1 (v/v) in cultures containing or lacking eSF, such that the final ratio of PBMCs:eSF was 10:1, as in the co-culture assay. Upon removal from eSF, PBMCs were transferred to new wells and immediately infected with 250 ng/ml p24^Gag^ NLENG1I. As a positive control, at the time of infection, PHA/IL2-activated T cells not pre-conditioned with eSF were added to eSF and infected with NLENG1I 250 ng/ml p24^Gag^. Infections were allowed to proceed for 3 d in the presence of 20 IU/ml IL-2 and then monitored for infection levels by flow cytometry.

### eEC leakage measurements

To determine the effects of estradiol and progesterone on endometrial epithelial monolayer permeability, eEC were cultured in 1-μm Millicell hanging cell culture inserts (EMD Millipore) coated with Matrigel (BD Biosciences) with donor-paired eSF in the basolateral chamber as described [14]. In all samples tested, eEC were confirmed to form an intact barrier prior to exposure to hormones as assessed by transepithelial resistance and leakage of phenol red (32 μg/ml) [14]. To monitor the effects of estradiol and progesterone on barrier function, the basolateral chambers of the cultures were then exposed to vehicle, 10 nM estradiol, or 10 nM estradiol + 1 μM progesterone for 6 days, with basolateral media replenished half way through the assay, similar to methods described [15, 57]. At the end of 6 d, 100 μl of apical and basolateral media were collected and assessed for absorbance at 559 nm using a Beckman Coulter DU 530 spectrophotometer (Beckman Coulter). Absorbance values of culture medium lacking phenol red served as background control, while absorbance values of culture media containing 32 μg/ml of phenol red served as the input control. For each donor, % leakage was calculated by the following formula: (OD_559_(basolateral)–OD_559_(background)) / ((OD_559_(input)—OD_559_(background)) x 100.

### eEC co-culture infection assays

To test the effect of eEC on HIV infection of CD4+ T cells, eEC cultured in 24-well matrigel plates were washed 4x with 1 ml RP2 medium (RPMI-1640 medium containing 2% FBS, penicillin (50 U/ml), streptomycin (50 μg/ml), and L-glutamine (2 mM)), and then replenished with 1 ml fresh RP2. Empty matrigel wells treated identically served as negative controls. After 24 h, 200 μl of medium was removed and served as eEC-conditioned medium. PHA/IL2-activated PBMC (10^5^/well) were then added directly to the eEC or matrigel wells containing medium supplemented with 10 IU/ml IL-2. Where indicated, activated PBMC were instead added to 1-μm transwells (Millipore). NLENG1I (15–100 ng/ml p24^Gag^) was added to the culture immediately following addition of PBMCs. To test the effects of eEC-conditioned medium on HIV infection of PBMCs, activated PBMCs were incubated with eEC- or mock-conditioned medium supplemented with 10 IU/ml IL-2 prior to infection. All infections were allowed to proceed for 3 d, at which time lymphocytes were harvested and stained for flow cytometric analyses.

### Flow cytometry

Cells were washed 1x in FACS buffer (PBS + 2% FBS + 2 mM EDTA) and then stained for 30 min at 4°C with fluorophore-conjugated antibodies in the presence of Zombie Aqua viability dye (Biolegend) diluted in FACS buffer. Antibodies used to identify CD4+ T cells were APC-H7-conjugated anti-CD3 (SK7), APC-conjugated anti-CD8 (SK1), and PE-Cy7-conjugated anti-CD4 (SK3). Antibodies used to assess activation state of CD4+ T cells were V450-conjugated anti-CD25 (M-A251) and PE-conjugated anti-HLA-DR (G46-6). Antibodies used for DC phenotyping were PE-conjugated anti-CD1a (HI149), FITC-conjugated anti-CD40 (5C3), and APC-conjugated CD83 (HB15e). Antibodies used to assess expression levels of co-receptors and adhesion molecules on eSF-exposed T cells were PE-conjugated anti-CCR5 (2D7/CCR5), PE-conjugated anti-CXCR4 (12G5), FITC-conjugated anti-CD11a (HI111), FITC-conjugated anti-CD18 (TS1/18), and PE-conjugated anti-CD54 (LB-2). eSF were identified by staining with PE-conjugated anti-CD140b (28D4). All antibodies were purchased from BD Biosciences except for the anti-CD11a and anti-CD18, which were purchased from Biolegend. After staining, cells were washed 2x with FACS buffer and then fixed with 2% paraformaldehyde for 24 h. Flow-count fluorospheres (Beckman Coulter) were used according to manufacturer’s protocol to determine the absolute numbers of GFP+ cells in each sample. Briefly, 5 μl of beads (at 1,000 beads/μl) were added to 45 μl of PFA-fixed cells immediately prior to analysis by flow cytometry, and normalization was carried out per manufacturer’s instructions. All flow cytometric samples were run on an LSR-II flow cytometer (BD Biosciences). Flow cytometric analyses were performed with FlowJo software (Treestar). All flow cytometric data with error bar measurements correspond to mean ± standard deviation.

### Microarray analysis

To determine which genes are most differentially expressed in eEC as compared to eSF, total RNA was processed from primary cultured eEC and eSF (n = 4 donors each), reverse transcribed into cDNA, labeled and hybridized to Affymetrix Human Gene 1.0 ST arrays as reported [56]. The resulting CEL files were analyzed using GeneSpring GX 11.02 software (Agilent) and processed using a robust multiarray analysis algorithm for background adjustment, normalization, and log2 transformation of perfect match values. RMA16 was utilized as the background correction algorithm for ST array technology. Differential expression of genes was outputted as fold-change increase against eSF (i.e. eEC-specific genes) calculated from a 2-way ANOVA with Benjamini–Hochberg multiple-testing correction for false discovery rate.

### Quantitation of anti-viral factors secreted by eEC

To quantitate the levels of anti-viral factors secreted by eEC, culture supernatants were collected from eEC after 48 h of culture. For each of three donors, 12 culture wells (1 ml/well) were set up. Quantitation was performed per manufacturer’s protocols using ELISA kits specific for secretory leukocyte protease inhibitor (SLPI) (R& D Systems), HBD1 (Alpha Diagnostics International), HBD2 (Alpha Diagnostics International), mucin-1 (Thermo Fisher Scientific), serpin A1 (Novus Biologicals), and serpin A3 (Novus Biologicals). For the mucin-1 ELISA, conversion of activity units into concentrations was calculated by assumption that each ng of mucin corresponds to 1.8 units antigen, based on the average mucin-1 serum concentration of 10 ng/ml mucin-1 and the detection of 18 U/ml of mucin-1 in serum [72].

### Statistics

Differences in infection rates between experimental conditions were assessed using ANOVA tests, followed by *t* tests to perform individual pairwise comparisons. Bonferroni adjustment was applied to account for multiple comparisons (one-way analysis of variance with a Bonferroni posttest). When two conditions were compared, a 2-tailed *t* test was used. Error bars on bar graphs correspond to experimental triplicate conditions, where three separate wells were treated identically throughout the entire course of the experiment.

### Ethics statement

Endometrial, cervical, foreskin, intestinal, and urethral tissues were obtained from UCSF and the Cooperative Human Tissue Network (CHTN) (IRB #s 10–07286, 14–15361, 10–01359, and 10–01218) in accordance with the Declaration of Helsinki after written informed consent. De-identified PBMCs were obtained from the Stanford Blood Bank under Exempt Status approved by the UCSF Committee of Human Research. Samples were only obtained from adults, who all provided written informed consent.

## Supporting information

S1 Fig eSF exhibit fibroblast morphology and express PDGFRB. (A) Phase-contrast image showing a confluent monolayer of primary eSF. Scale bar corresponds to 250 μm.
(B) Flow cytometric assessment of cell-surface expression of the eSF marker PDGFRB. Gated on live, singlet events.
(EPS)

S2 Fig eSF enhance HIV infection of naturally permissive tissue-derived cells. CD19-depleted cells isolated from human tonsils from 2 donors were infected with NLENG1I, in the absence or presence of eSF. Infection levels were monitored 3 d later by flow cytometry. Results are gated on live, singlet CD3+CD8- cells, and are representative of four experiments with different eSF donors. **p<0.01 (by 2-tailed *t* test).
(EPS)

S3 Fig eSF-mediated enhancement of HIV infection of CD4+ T cells is most potent at low viral inocula. (A) PHA/IL2-activated PBMCs cultured with or without eSF were infected with the indicated concentrations of NLENG1I and monitored by flow cytometry for infection levels 3 d later. Numbers in green correspond to fold-enhancement of HIV infection.
(B) Enhancement of viral infection rates can reach up to ~100-fold when baseline level of infection in the absence of eSF is minimal. PHA/IL2-activated PBMCs cultured with or without eSF from 3 different donors were monitored for infection levels 3 d later. Results are gated on live, singlet CD3+CD8- cells.
(EPS)

S4 Fig eSF-mediated enhancement of R5-tropic HIV infection T cells occurs under low vial inoculum conditions, and with purified CD4+ T cells activated by either PHA/IL2 or anti-CD3/CD28. (A) PHA/IL2-activated PBMCs cultured with or without eSF were infected with limiting amounts of BaL_GFP_ and monitored by flow cytometry for infection levels 3 d later. Results are gated on live, singlet CD3+CD8- cells.
(B) PBMC-derived CD4+ T cells were isolated to >96% purity for experiments described in Panels *C* and *D*.
(C) The purified CD4+ T cells shown in panel *B* were activated with PHA/IL2 or anti-CD3/CD28 and then infected with BaL_GFP_. Infection levels were monitored 3 d later by flow cytometry. Results are gated on live, singlet CD3+CD8- cells. ***p<0.001, ****p<0.0001 relative to no co-culture in a group-wise comparison (one-way analysis of variance with a Bonferroni posttest).
(D) As in panel *C*, except that the T/F strain THRO_GFP_ was used instead of BaL_GFP_.
(EPS)

S5 Fig eSF enhance the numbers of HIV-infected CD4+ T cells in culture. (A) PHA/IL2-activated PBMCs from 2 donors were infected with NLENG1I in the absence or presence of eSF and monitored for absolute numbers of HIV-infected cells 3 d later by flow cytometry. Results correspond to live, singlet CD3+CD8-GFP+ cells.
(B) PHA/IL2- or CD3/CD28-activated PBMCs were infected with an HIV reporter virus encoding Firefly luciferase (NL4-3.Luc) with or without eSF, and monitored for infection levels 3 d later by luminescence. **p<0.01, ***p<0.001 (by 2-tailed *t* test).
(EPS)

S6 Fig eSF do not activate CD4+ T cells and eSF-mediated enhancement occurs with autologous T cells. (A) Unstimulated or PHA/IL2-activated CD4+ T cells were cultured in the absence or presence of eSF for 3 d, and then monitored for cell-surface levels of the activation markers CD25 (*left*) and HLA-DR (*right*). The activation profiles of T cells in the absence (*black*) and presence (*red*) of eSF largely overlay. PHA/IL2 upregulates both CD25 and HLA-DR expression, as expected, but this level is not further increased with eSF co-culture. Results are gated on live, singlet CD3+CD8- cells.
(B) Unstimulated (No Stim) or activated (PHA or PHA/IL2) PBMCs from four donors were co-cultured with autologous or heterologous eSF and infected with NLENG1I. Infection levels were monitored 3 d later by flow cytometry. *Right*: No Stim data are shown on a smaller scale to demonstrate the low level of eSF-mediated enhancement of infection of unstimulated CD4+ T cells.
(EPS)

S7 Fig Effect of HeLa and T ESCs cell lines on HIV infection of CD4+ T cells. (A) Unstimulated or PHA/IL2-activated PBMCs cultured with or without eSF or HeLa were infected with NLENG1I and monitored for infection levels 3 d later. Results are gated on live, singlet CD3+CD8- cells.
(B) Activated PBMCs cultured with or without T ESCs or eSF were infected with NLENG1I and monitored for infection levels 3 d later. Results are gated on live, singlet CD3+CD8- cells, and representative FACS plots of triplicate conditions are shown. ***p<0.001 and ****p<0.0001 relative to No co-culture control in a group-wise comparison (one-way analysis of variance with a Bonferroni posttest). Results are representative of one of two experiments conducted.
(EPS)

S8 Fig eSF-mediated enhancement of HIV infection is not due to productive infection of eSF. (A) PBMCs co-cultured with eSF were inoculated with NLENG1I (*left*) or BaL_GFP_ (*right*) and both cell types were assessed for productive infection after 3 d.
(B) Unstimulated or activated PBMCs cultured with or without eSF were infected with replication-incompetent NL4-3.env*GFP, and monitored for infection levels 3 d later by flow cytometry. Results are gated on live, singlet CD3+CD8- cells.
(C) PHA/IL2-activated PBMCs cultured with or without eSF from 3 different donors were infected with replication-incompetent Nef-deficient R5-tropic HIV and monitored for infection levels 3 d later by flow cytometry. Results are gated on live, singlet CD3+CD8- cells.
****p<0.0001 relative to No co-culture control in a group-wise comparison (one-way analysis of variance with a Bonferroni posttest). *Left*: Results of experimental triplicate measurements. *Right*: Representative flow cytometric plots.
(EPS)

S9 Fig Kinetics of HIV infection of T cells in the absence and presence of eSF. PHA/IL2-activated PBMCs cultured with or without eSF were infected with NLENG1I (A) or BaL_GFP_ (B) and monitored for infection levels at the indicated timepoints. Results are gated on live, singlet CD3+CD8- cells. The proportions of HIV-infected cells are displayed on the left, while the absolute numbers of HIV-infected cells are displayed on the right. *p<0.05, **p<0.01, and ****p<0.0001 in group-wise comparisons (one-way analysis of variance with a Bonferroni posttest). n.s.: non-significant. Numbers underneath each colored bar correspond to fold-enhancement over the “No eSF” condition for each timepoint.
(EPS)

S10 Fig eSF enhance fusion of HIV to CD4+ T cells. PBMCs infected with BlaM-encoding X4-tropic NL4-3 (A) or R5-tropic BaL (B) with or without eSF were monitored for viral fusion levels. *Top*: Representative flow cytometric plots. *Bottom*: Results of experimental triplicate measurements. **p<0.01, ***p<0.001 (by 2-tailed *t* test). Shown are one of two representative experiments with different eSF donors.
(EPS)

S11 Fig Characterization of HIV attachment to eSF and DCs. (A) Expression of DC and activation markers on iDCs and mDCs. Cells were analyzed by flow cytometry for the DC markers CD40 and CD1a, or the DC maturation marker CD83.
(B) Attachment of HIV to eSF and DCs. Equal number of the indicated cells were incubated with virus for 2 h at 37°C, washed 4x, and then assessed by p24 ELISA for levels of bound virus.
(C) eSF or DCs were assessed by flow cytometry for cell-surface expression of the *trans*-infection receptors DC-SIGN or Siglec-1. iDCs served as a positive control for DC-SIGN staining, while mDCs served as a positive control for Siglec-1 staining.
(D) Mannan does not inhibit *trans*-infection to CD4+ T cells. iDCs, RAJI-DC-SIGN, or eSF were pre-treated with mannan (50 μg/ml), pulsed with NLENG1I, and incubated with PHA/IL2-activated PBMCs, and infection levels were monitored 3 d later and reported as percentage inhibition relative to the corresponding cell type not treated with mannan. ****p<0.0001 relative to no mannan control in a group-wise comparison (one-way analysis of variance with a Bonferroni posttest) for each cell type.
(E) PHA/IL2-activated PBMCs cultured with mock- or heparinase-treated eSF were infected with NLENG1I and then monitored for infection levels 3 d later. The panel on the left shows the levels of cell-surface HSPG in heparinase-treated eSF, gated on live, singlet cells. The panel on the right shows the ability of mock- and heparinase-treated eSF to enhance HIV infection of T cells, and is gated on live, singlet CD3+CD8- cells.
(EPS)

S12 Fig Effect of eSF on cell-surface levels of HIV-1 receptor, HIV-1 co-receptors, and adhesion molecules on T cells. PHA/IL2-activated T cell were co-cultured for 3 d with eSF, and then assessed for cell-surface levels of CD4 or the co-receptors CXCR4 and CCR5 (A), or the adhesion molecules CD11a (α-chain component of LFA-1), CD18 (β-chain component of LFA-1) and CD54 (ICAM-1) (B). Unstained samples correspond to Fluorescence Minus One (FMO) controls. Results are gated on live, singlet CD3+CD8- cells, and are representative of one of two experiments using different sets of eSF and PBMC donors.
(EPS)

S13 Fig Comparison of HIV infection enhancement by eSF and 10-fold excess DCs. PHA/IL2-activated PBMCs were infected with replication-competent NLENG1I (A) or replication-incompetent NL4-3.env*GFP (B) in isolation, or in the presence of eSF, an equal number of DCs, or 10x the amount of DCs. Infection levels were monitored 3 d later by flow cytometry. Results are gated on live, singlet CD3+CD8- cells. *p<0.05, ***p<0.001, and ****p<0.0001 relative to No co-culture control in a group-wise comparison (one-way analysis of variance with a Bonferroni posttest).
(EPS)

S14 Fig Stromal fibroblasts isolated from endometrium, ectocervix, and endocervix of the same subject enhance HIV infection of CD4+ T cells to similar extents. PHA/IL2-activated PBMCs were infected with NLENG1I in isolation, or in the presence of eSF, cSF, or cnSF. Infection levels were monitored 3 d later by flow cytometry. Results are gated on live, singlet CD3+CD8- cells. **p<0.01 and ***p<0.001 in a group-wise comparison (one-way analysis of variance with a Bonferroni posttest) of all samples.
(EPS)

S15 Fig Fibroblasts do not proliferate over the course of co-culture. eSF were seeded in 96-well flat-bottom tissue culture plates and cultured until confluency, at which time cells were removed by trypsin/EDTA and counted with a hemocytometer. A separate set of wells were counted three days after cells achieved confluency.
(EPS)

S16 Fig Gating strategy used for determining percentage of HIV-infected CD4+ T cells. PBMCs were gated on live cells through use of an amine-reactive dye (Zombie Aqua), followed by gating on singlets. CD4+ T cells were identified by gating on CD3+CD8- cells. Productively infected cells are in large part CD4- because of HIV-mediated CD4 downregulation.
(EPS)

S1 Table Expression of anti-HIV factors is highly enriched in eEC compared to eSF. Shown are the top 40 genes more highly expressed in eEC than eSF as assessed by Genespring. Highlighted genes encode secreted proteins with known anti-HIV activity.
(PPTX)
