# Supplementary material for: Mucosal stromal fibroblasts markedly enhance HIV infection of CD4+ T cells
Source: PLoS Pathog. 2017 Feb 16;13(2):e1006163. doi: 10.1371/journal.ppat.1006163 (PMC5312882; doi:10.1371/journal.ppat.1006163)
Supplement: S1 Table — Shown are the top 40 genes more highly expressed in eEC than eSF as assessed by Genespring. Highlighted genes encode secreted proteins with known anti-HIV activity. (PPTX) [file ppat.1006163.s017.pptx]

## Slide 1
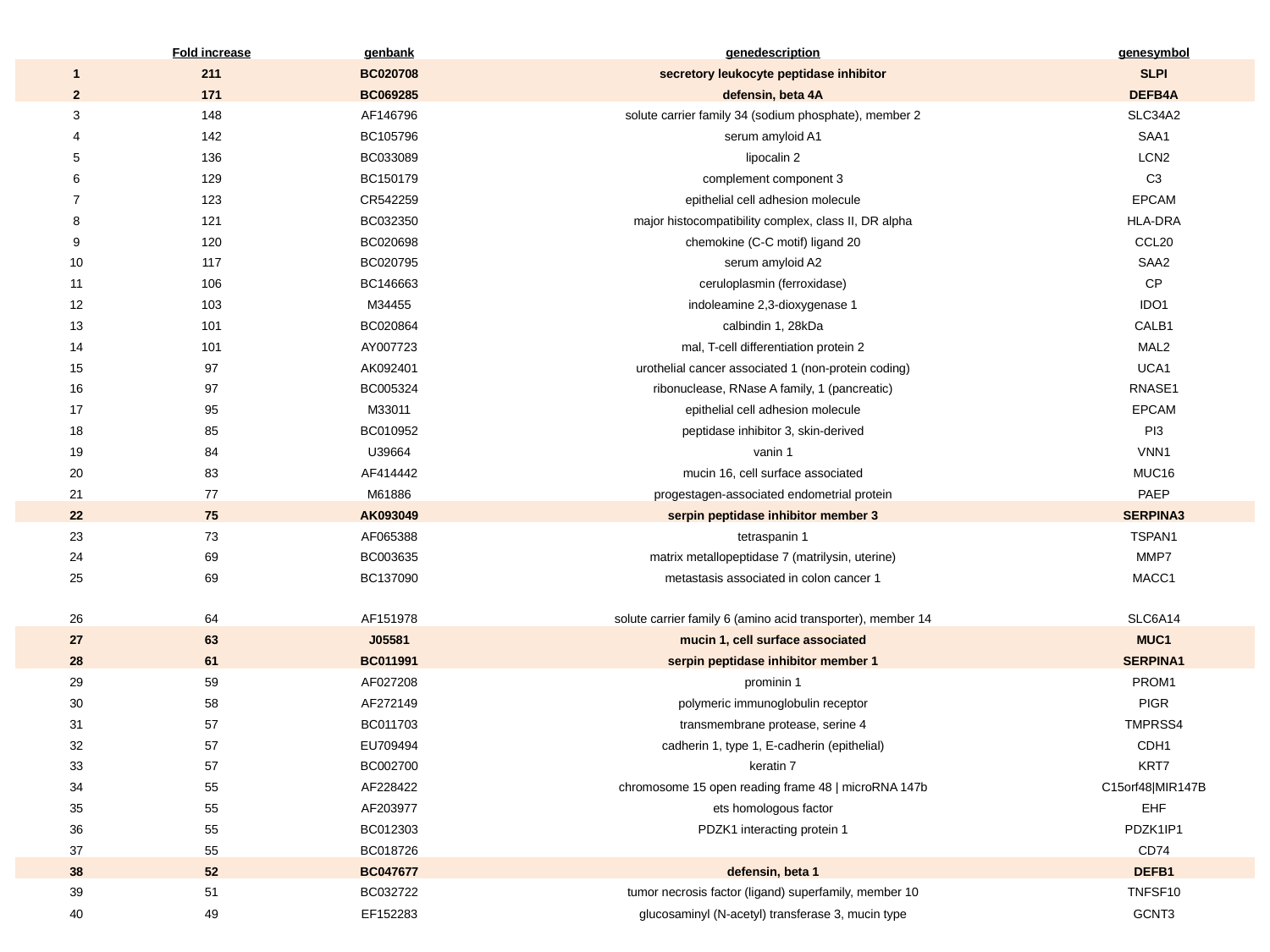

| | Fold increase | genbank | genedescription | genesymbol |
| --- | --- | --- | --- | --- |
| 1 | 211 | BC020708 | secretory leukocyte peptidase inhibitor | SLPI |
| 2 | 171 | BC069285 | defensin, beta 4A | DEFB4A |
| 3 | 148 | AF146796 | solute carrier family 34 (sodium phosphate), member 2 | SLC34A2 |
| 4 | 142 | BC105796 | serum amyloid A1 | SAA1 |
| 5 | 136 | BC033089 | lipocalin 2 | LCN2 |
| 6 | 129 | BC150179 | complement component 3 | C3 |
| 7 | 123 | CR542259 | epithelial cell adhesion molecule | EPCAM |
| 8 | 121 | BC032350 | major histocompatibility complex, class II, DR alpha | HLA-DRA |
| 9 | 120 | BC020698 | chemokine (C-C motif) ligand 20 | CCL20 |
| 10 | 117 | BC020795 | serum amyloid A2 | SAA2 |
| 11 | 106 | BC146663 | ceruloplasmin (ferroxidase) | CP |
| 12 | 103 | M34455 | indoleamine 2,3-dioxygenase 1 | IDO1 |
| 13 | 101 | BC020864 | calbindin 1, 28kDa | CALB1 |
| 14 | 101 | AY007723 | mal, T-cell differentiation protein 2 | MAL2 |
| 15 | 97 | AK092401 | urothelial cancer associated 1 (non-protein coding) | UCA1 |
| 16 | 97 | BC005324 | ribonuclease, RNase A family, 1 (pancreatic) | RNASE1 |
| 17 | 95 | M33011 | epithelial cell adhesion molecule | EPCAM |
| 18 | 85 | BC010952 | peptidase inhibitor 3, skin-derived | PI3 |
| 19 | 84 | U39664 | vanin 1 | VNN1 |
| 20 | 83 | AF414442 | mucin 16, cell surface associated | MUC16 |
| 21 | 77 | M61886 | progestagen-associated endometrial protein | PAEP |
| 22 | 75 | AK093049 | serpin peptidase inhibitor member 3 | SERPINA3 |
| 23 | 73 | AF065388 | tetraspanin 1 | TSPAN1 |
| 24 | 69 | BC003635 | matrix metallopeptidase 7 (matrilysin, uterine) | MMP7 |
| 25 | 69 | BC137090 | metastasis associated in colon cancer 1 | MACC1 |
| 26 | 64 | AF151978 | solute carrier family 6 (amino acid transporter), member 14 | SLC6A14 |
| 27 | 63 | J05581 | mucin 1, cell surface associated | MUC1 |
| 28 | 61 | BC011991 | serpin peptidase inhibitor member 1 | SERPINA1 |
| 29 | 59 | AF027208 | prominin 1 | PROM1 |
| 30 | 58 | AF272149 | polymeric immunoglobulin receptor | PIGR |
| 31 | 57 | BC011703 | transmembrane protease, serine 4 | TMPRSS4 |
| 32 | 57 | EU709494 | cadherin 1, type 1, E-cadherin (epithelial) | CDH1 |
| 33 | 57 | BC002700 | keratin 7 | KRT7 |
| 34 | 55 | AF228422 | chromosome 15 open reading frame 48 | microRNA 147b | C15orf48|MIR147B |
| 35 | 55 | AF203977 | ets homologous factor | EHF |
| 36 | 55 | BC012303 | PDZK1 interacting protein 1 | PDZK1IP1 |
| 37 | 55 | BC018726 | | CD74 |
| 38 | 52 | BC047677 | defensin, beta 1 | DEFB1 |
| 39 | 51 | BC032722 | tumor necrosis factor (ligand) superfamily, member 10 | TNFSF10 |
| 40 | 49 | EF152283 | glucosaminyl (N-acetyl) transferase 3, mucin type | GCNT3 |
